# Supplementary material for: Incidence of HBV Reactivation in Psoriasis Patients Undergoing Cytokine Inhibitor Therapy: A Single-Center Study and Systematic Review with a Meta-Analysis
Source: Viruses. 2024 Dec 30;17(1):42. doi: 10.3390/v17010042 (PMC11769189; doi:10.3390/v17010042)

Table S1. PRISMA 2020 checklist.

Section and Topic	Item #	Checklist item	Location where item is reported
**TITLE**			
Title	1	Identify the report as a systematic review.	Page 1
**ABSTRACT**			
Abstract	2	See the PRISMA 2020 for Abstracts checklist.	NA
**INTRODUCTION**			
Rationale	3	Describe the rationale for the review in the context of existing knowledge.	Page 5
Objectives	4	Provide an explicit statement of the objective(s) or question(s) the review addresses.	Page 5
**METHODS**			
Eligibility criteria	5	Specify the inclusion and exclusion criteria for the review and how studies were grouped for the syntheses.	Page 7
Information sources	6	Specify all databases, registers, websites, organisations, reference lists and other sources searched or consulted to identify studies. Specify the date when each source was last searched or consulted.	Page 7
Search strategy	7	Present the full search strategies for all databases, registers and websites, including any filters and limits used.	Table S2
Selection process	8	Specify the methods used to decide whether a study met the inclusion criteria of the review, including how many reviewers screened each record and each report retrieved, whether they worked independently, and if applicable, details of automation tools used in the process.	Protocol (INPLASY202490005)
Data collection process	9	Specify the methods used to collect data from reports, including how many reviewers collected data from each report, whether they worked independently, any processes for obtaining or confirming data from study investigators, and if applicable, details of automation tools used in the process.	Protocol (INPLASY202490005)
Data items	10a	List and define all outcomes for which data were sought. Specify whether all results that were compatible with each outcome domain in each study were sought (e.g. for all measures, time points, analyses), and if not, the methods used to decide which	Table S5

Section and Topic	Item #	Checklist item	Location where item is reported
		results to collect.	
	10b	List and define all other variables for which data were sought (e.g. participant and intervention characteristics, funding sources). Describe any assumptions made about any missing or unclear information.	Table S5
Study risk of bias assessment	11	Specify the methods used to assess risk of bias in the included studies, including details of the tool(s) used, how many reviewers assessed each study and whether they worked independently, and if applicable, details of automation tools used in the process.	Table S5
Effect measures	12	Specify for each outcome the effect measure(s) (e.g. risk ratio, mean difference) used in the synthesis or presentation of results.	Table 2
Synthesis methods	13a	Describe the processes used to decide which studies were eligible for each synthesis (e.g. tabulating the study intervention characteristics and comparing against the planned groups for each synthesis (item #5)).	Protocol (INPLASY202490005)
	13b	Describe any methods required to prepare the data for presentation or synthesis, such as handling of missing summary statistics, or data conversions.	NA
	13c	Describe any methods used to tabulate or visually display results of individual studies and syntheses.	NA
	13d	Describe any methods used to synthesize results and provide a rationale for the choice(s). If meta-analysis was performed, describe the model(s), method(s) to identify the presence and extent of statistical heterogeneity, and software package(s) used.	Page 10
	13e	Describe any methods used to explore possible causes of heterogeneity among study results (e.g. subgroup analysis, meta-regression).	Page 10
	13f	Describe any sensitivity analyses conducted to assess robustness of the synthesized results.	Page 10
Reporting bias assessment	14	Describe any methods used to assess risk of bias due to missing results in a synthesis (arising from reporting biases).	Page 10
Certainty assessment	15	Describe any methods used to assess certainty (or confidence) in the body of evidence for an outcome.	NA
**RESULTS**			

Section and Topic	Item #	Checklist item	Location where item is reported
Study selection	16a	Describe the results of the search and selection process, from the number of records identified in the search to the number of studies included in the review, ideally using a flow diagram.	Figure S1
	16b	Cite studies that might appear to meet the inclusion criteria, but which were excluded, and explain why they were excluded.	Table S4
Study characteristics	17	Cite each included study and present its characteristics.	Table S5
Risk of bias in studies	18	Present assessments of risk of bias for each included study.	Table S5
Results of individual studies	19	For all outcomes, present, for each study: (a) summary statistics for each group (where appropriate) and (b) an effect estimate and its precision (e.g. confidence/credible interval), ideally using structured tables or plots.	Table 2
Results of syntheses	20a	For each synthesis, briefly summarise the characteristics and risk of bias among contributing studies.	Page 12
	20b	Present results of all statistical syntheses conducted. If meta-analysis was done, present for each the summary estimate and its precision (e.g. confidence/credible interval) and measures of statistical heterogeneity. If comparing groups, describe the direction of the effect.	Table 2
	20c	Present results of all investigations of possible causes of heterogeneity among study results.	NA
	20d	Present results of all sensitivity analyses conducted to assess the robustness of the synthesized results.	Figure S4
Reporting biases	21	Present assessments of risk of bias due to missing results (arising from reporting biases) for each synthesis assessed.	Figure S3
Certainty of evidence	22	Present assessments of certainty (or confidence) in the body of evidence for each outcome assessed.	NA
**DISCUSSION**			
Discussion	23a	Provide a general interpretation of the results in the context of other evidence.	Page 12
	23b	Discuss any limitations of the evidence included in the review.	Page 16

Section and Topic	Item #	Checklist item	Location where item is reported
	23c	Discuss any limitations of the review processes used.	Page 16
	23d	Discuss implications of the results for practice, policy, and future research.	Page 16
**OTHER INFORMATION**			
Registration and protocol	24a	Provide registration information for the review, including register name and registration number, or state that the review was not registered.	Protocol (INPLASY202490005)
	24b	Indicate where the review protocol can be accessed, or state that a protocol was not prepared.	Protocol (INPLASY202490005)
	24c	Describe and explain any amendments to information provided at registration or in the protocol.	NA
Support	25	Describe sources of financial or non-financial support for the review, and the role of the funders or sponsors in the review.	Page 2
Competing interests	26	Declare any competing interests of review authors.	Page 1
Availability of data, code and other materials	27	Report which of the following are publicly available and where they can be found: template data collection forms; data extracted from included studies; data used for all analyses; analytic code; any other materials used in the review.	NA

Table S2. Search strategy.

Database	Keywords	Date	Results
PubMed	(Psoriasis OR Psoriasis vulgaris OR Psoriatic arthropathy) AND ((interleukin 17 OR ixekizumab OR secukinumab OR brodalumab) OR (interleukin 23 OR Risankizumab) OR (interleukin12/23 OR Ustekinumab)) AND HBV reactivation	2024.07.02	20
Embase			86
Cochrane Central Register of Controlled Trials			1
Web of Science			21

Table S3. Clinical features of psoriasis patients who developed HBVr during cytokine inhibitor therapy.

	HBcAb –/**HBcAb+**	
**Data source**	**Case 1**	**Case 2**
Age/sex	79/F	63/M
Psoriasis disease duration (year)	10	20
Duration from cytokine therapy to HBVr (drug, dose)	Brodalumab (3)	Ustekinumab (5)
HBsAg seroreversion	N	Y
HBsAb	N	NA
Anti-HCV	N	Y
HBV DNA (initial/peak, IU/mL)	ND/< 10	ND /$2.8X10^{7}$
ALT (initial/peak, U/L)	12/16	11/165
T.bil (initial/peak, mg/dL)	NA/NA	NA/18.1
Hepatitis flare-up	NA	Y
Treatment for HBVr	N	ETV 0.5 mg
Outcome	Alive & well	Expire
sDMARDs	MTX	MTX/CSA
BDMARDs before (drug, dose)	Etanercept (502)	Secukinumab (7)

HBVr, hepatitis B virus reactrivation; HBsAg, HBV surface antigen; HBc Ab, HBV core antibody; F, female; M, male; HCV, hepatitis C virus; ALT, alanine aminotransferase; T.bil, total bilirubin; ETV, Entecavir; TAF, tenofovir alafenamide fumarate; sDMARDs, synthetic disease-modifying antirheumatic drugs; MTX, methotrexate; CSA, cyclosporine; NA, not applicable; N, did not happen; Y, happened.

Table S4. Excluded studies and reasons for exclusion

1. Case numbers less than 5

2. Lacked HBV status reporting

3. Overlapping populations

Citation	Reasons
Navarro R, Vilarrasa E, Herranz P, Puig L, Bordas X, Carrascosa JM, Taberner R, Ferrán M, García-Bustinduy M, Romero-Maté A, Pedragosa R, García-Diez A, Daudén E. Safety and effectiveness of ustekinumab and antitumour necrosis factor therapy in patients with psoriasis and chronic viral hepatitis B or C: a retrospective, multicentre study in a clinical setting. Br J Dermatol. 2013 Mar;168(3):609-16. doi: 10.1111/bjd.12045. PMID: 22985451.	1
Siegel S.A.R.; Winthrop K.L.; Ehst B.D.; Ortega-Loayza A.G.Secukinumab treatment of individuals with psoriasis infected with hepatitis B and/or hepatitis C virus	1
Solay AH, Acar A, Eser F, Kuşcu F, Tütüncü EE, Kul G, Şentürk GÇ, Gürbüz Y. Reactivation rates in patients using biological agents, with resolved HBV infection or isolated anti-HBc IgG positivity. Turk J Gastroenterol. 2018 Sep;29(5):561-565. doi: 10.5152/tjg.2018.18032. PMID: 30260778; PMCID: PMC6284614.	1
Özçelik S.; Kılıç F.A.Hepatitis B virus reactivation in patients with psoriasis on biologic therapies: A retrospective study.DOI 10.4103/TJD.TJD_42_20	1
Hung MH, Tien YC, Chiu YM. Risk factors for losing hepatitis B virus surface antibody in patients with HBV surface antigen negative/surface antibody positive serostatus receiving biologic disease-modifying anti-rheumatic drugs: a nested case-control study. Adv Rheumatol. 2021 Apr 8;61(1):22. doi: 10.1186/s42358-021-00173-9. PMID: 33832541.	1
Wang J, Geng X, Zhang X, Xiao Y, Wang W. Hepatitis B Virus Reactivation and Mycobacterial Infections Associated With Ustekinumab: A Retrospective Study of an International Pharmacovigilance Database. Front Pharmacol. 2022 Jul 4;13:921084. doi: 10.3389/fphar.2022.921084. PMID: 35860015; PMCID: PMC9289361.	2
Chiu HY, Chiu YM, Chang Liao NF, Chi CC, Tsai TF, Hsieh CY, Hsieh TY, Lai KL, Chiu TM, Wu NL, Hui RC, Lee CN, Wang TS, Chen PH, Yang CC, Huang YH. Predictors of hepatitis B and C virus reactivation in patients with psoriasis treated with biologic agents: a 9-year multicenter cohort study. J Am Acad Dermatol. 2021 Aug;85(2):337-344. doi: 10.1016/j.jaad.2019.12.001. Epub 2019 Dec 9. PMID: 31821860.	2
Kridin K, Zirpel H, Mruwat N, Ludwig RJ, Thaci D. Evaluating the risk of infections under interleukin 23 and interleukin 17 inhibitors relative to tumour necrosis factor inhibitors - A population-based study. J Eur Acad Dermatol Venereol. 2023 Nov;37(11):2319-2326. doi: 10.1111/jdv.19328. Epub 2023 Jul 19. PMID: 37466275.	2
Chiu HY, Hui RC, Huang YH, Huang RY, Chen KL, Tsai YC, Lai PJ, Wang TS, Tsai TF. Safety Profile of Secukinumab in Treatment of Patients with Psoriasis and Concurrent Hepatitis B or C: A Multicentric Prospective Cohort Study. Acta Derm Venereol. 2018 Oct 10;98(9):829-834. doi: 10.2340/00015555-2989. PMID: 29972221.	3

Table S5. Demographic and characteristics of included studies.

A. HBsAg+ patients without prophylactics (n = 25).

First Author (year)	Location	Setting/ city	Study design	Age (years)	Drug catalog/ Drug	Dosage	Follow- up	Definition of HBVr	HBVr (n/N)	HBVr time	HBVr associated hepatitis/ death	ROB by NOS
Chiu 2013	Taiwan	S/Taipei	R	46#	interleukin12/23 (ustekinumab)	standard dose%	9.43# (4-39) months	HBV DNA reappearance or elevation	2/7	4 months, 7 months	0/0	High
Ting 2018	Taiwan	M/Taipei and Linkou	R	44# ± 9	interleukin12/23 (ustekinumab)	standard dose%	24# ± 12 months	HBV DNA reappearance or elevation	2/8	3 months, 6 months	0/0	Mode-rate
Qin 2022	China	S/Shanghai	R	43.7#	interleukin 17 (secukinumab)	standard dose%	24 weeks	HBV DNA reappearance or elevation	0/2	Nil	0/0	High
Lu 2024	China	M/Anhui	R	42#	interleukin12/23 (ustekinumab)	standard dose%	28 weeks	HBV DNA load increase more than 100-fold compared with baseline; detectable HBV DNA in patients previously with undetectable DNA;	0/3	Nil	0/0	High
Present study	Taiwan	S/chiayi	R	51#	interleukin 17 (Ixekizumab, Secukinumab, Brodalumab)	standard dose%	5.3# (0.8-10.8) years	HBV DNA > 2log over baseline or > 3log with previously undetectable or > 4log if baseline level was unavailable	0/5	Nil	0/0	High

B. HBsAg+ patients with prophylactics (n = 31).

First Author (year)	Location	Setting/ city	Study design	Age (years)	Drug catalog/ Drug	Dosage	Follow- up	Definition of HBVr	HBVr (n/N)	ROB by NOS
Chiu 2013	Taiwan	S/Taipei	R	46#	interleukin12/23 (ustekinumab)	Standard dose[%]	9.43* (4-39) months	HBV DNA reappearance or elevation	0/4	High
Ting 2018	Taiwan	M/Taipei and Linkou	R	44 ±9*	interleukin12/23 (ustekinumab)	standard dose[%]	24 ± 12*[&] months	HBV DNA reappearance or elevation	0/2	Mode-rate
AlMutairi 2018	Kuwait	S/Kuwait	P	51#	interleukin12/23 (ustekinumab)	NR	41 ± 21 months	HBV DNA reappearance or elevation	0/4	High
Megna 2022	Italy	M/seven centers	R	59.3#	interleukin 17 (secukinumab)	standard dose[%]	53.5 ± 37.5 weeks (range 16–240 weeks)	HBV DNA reappearance or elevation	0/13	High
Qin 2022	China	S/Shanghai	R	43.7#	interleukin 17 (secukinumab)	standard dose[%]	24 weeks	HBV DNA reappearance or elevation	0/2	High
Present study	Taiwan	S/chiayi	R	51#	interleukin12/23 (ustekinumab)	standard dose%	5.3# (0.8-10.8) years	HBV DNA > 2log over baseline or > 3log with previously undetectable or > 4log if baseline level was unavailable	0/2	High
					interleukin 17 (Ixekizumab, Secukinumab, Brodalumab)				0/4	High

C. HBsAg−/ HBcAb+ patients without prophylaxis (n = 218).

First Author (year)	Location	Setting/ city	Study design	Age (years)	Drug catalog/ Drug	Dosage	Follow- up	Definition of HBVr	HBVr, n/N	HBsAb+	HBsAb-	HBVr time	HBVr associated hepatitis/ death	ROB by NOS
Chiu 2013	Taiwan	S/Taipei	R	46#	interleukin12/23 (ustekinumab)	Standard dose%	9.43* (4-39) months	HBV DNA reappearance or elevation	0/3	2	1	NIL	0/0	High
Snast 2017	Israel	S/Israel	R	52.2#	Interleukin 12/23 (ustekinumab)	NR	4.45 years	HBV DNA reappearance or elevation	0/9	5	3	NIL	0/0	High
					interleukin 17 (secukinumab)				0/3	2	1			
AlMutairi 2018	Kuwait	S/Kuwait	P	51#	interleukin12/23 (ustekinumab)	NR	41 ± 21 months	HBV DNA reappearance or elevation	0/28	NR	NR	NIL	0/0	High
Ting 2018	Taiwan	M/Taipei and Linkou	R	45*	interleukin12/23 (ustekinumab)	standard dose%	23.4 *(5-44) months	HBV DNA reappearance or elevation	1/44	38	6	12 months	0/0	Mode -rate
Qin 2022	China	S/Shanghai	R	43.7#	interleukin 17 (secukinumab)	standard dose%	24 weeks	HBV DNA reappearance or elevation	0/15	14	1	NIL	0/0	High
Klujszo 2022	Poland	S/Kielce	R	63#	interleukin12/23 (ustekinumab)	NR	45 (30-178) weeks	HBV DNA reappearance	0/5	4	1	NIL	0/0	High
Megna 2022	Italy	M/seven centers	R	59.3#	interleukin 17 (secukinumab)	standard dose%	53.5 ± 37.5 weeks (range 16–240 weeks)	HBV DNA reappearance or elevation	1/17	NR	NR	12 months	1/0	High

Gargiulo 2022	Italy	S/Milan	R	NR	interleukin12/23 (ustekinumab)	NR	104 weeks	(1) HBV DNA reappearance or elevation (2) HBsAg seroreversion	0/1	1	0	0	0/0	High
					interleukin 17 (brodalumab secukinumab ixekizumab)				0/4	1	3			
					interleukin 23 (risankizumab)				0/9	2	7			
Lu 2024	China	M/Anhui	R	42#	interleukin12/23 (ustekinumab)	standard dose%	28 weeks	positive HBsAg in patients previously with negative HBsAg as well as liver dysfunction defined as threefold increase in ALT compared with baseline or absolute value of ALT level more than 100 U/L	0/18	10	NR	NIL	0/0	High
Present study	Taiwan	S/chiayi	R	51#	interleukin12/23 (ustekinumab)	standard dose%	5.3# (0.8-10.8) years	HBsAg seroconversion	1/7	4	2	10 months	1/1	High
					interleukin 17 (Ixekizumab, Secukinumab, Brodalumab)				1/52	37	8	2 months	0/1	
					interleukin 23 (risankizumab)				0/3	1	2	NIL	0/0	

* median

# mean

[%] standard dose:

interleukin12/23_Ustekinumab: 45 mg at weeks 0 and 4, followed by a maintenance dose once every 2–3 months;

interleukin 17_Secukinumab: 150 or 300 mg subcutaneously at week 0, 1, 2, 3, and 4, then 300 mg every 4 weeks;

interleukin 17_Brodalumab:210 mg subcutaneously at week 0,1,2, then 210 mg every 2 weeks;

interleukin 17_Ixekizumab: 300 mg subcutaneously at week 0, 1, 2, 3, and 4, then 300 mg every 4 weeks;

interleukin 23_ risankizumab: 150 mg subcutaneously at week 0,4, then 150 mg every 12 weeks.

HBVr, HBV reactivation; ROB, risk of bias; S, single-center; M, multi-center; P, prospective; R, retrospective; HBV, hepatitis B virus; HBsAg, hepatitis B surface antigen; ROB, risk of bias; Anti-HBs, Anti-Hepatitis B surface antibody; NOS, Newcastle–Ottawa scale; NR, data not reported; NIL, data do not need to report

Table S6. Subgroup analysis of the pooled rates of HBVr in HBsAg−/ HBcAb+ patients with HBsAb- compared to with HBsAb+ group.

Subgroup	N of Records	HBsAb-		HBsAb+		Risk difference	95% confidence interval	I^2(%)
		Event	Total	Event	Total			
**Overall**	12	2	35	1	111	0.04	-0.08 – 0.16	0
**Drug catalog**								
Interleukin 12/23 inhibitor	6	1	13	1	54	0.02	-0.15 – 0.18	0
Interleukin 17 inhibitor	4	1	13	0	54	0.09	-0.12 – 0.30	NA
Interleukin 23 inhibitor	2	0	9	0	3	0.00	-0.39 – 0.39	NA
**Study Region**								
Asian	6	2	20	1	96	0.05	-0.09 – 0.19	0
Non- Asian	6	0	15	0	15	0.00	-0.24 – 0.24	0

Figure S1. PRISMA diagram of literature search and selection

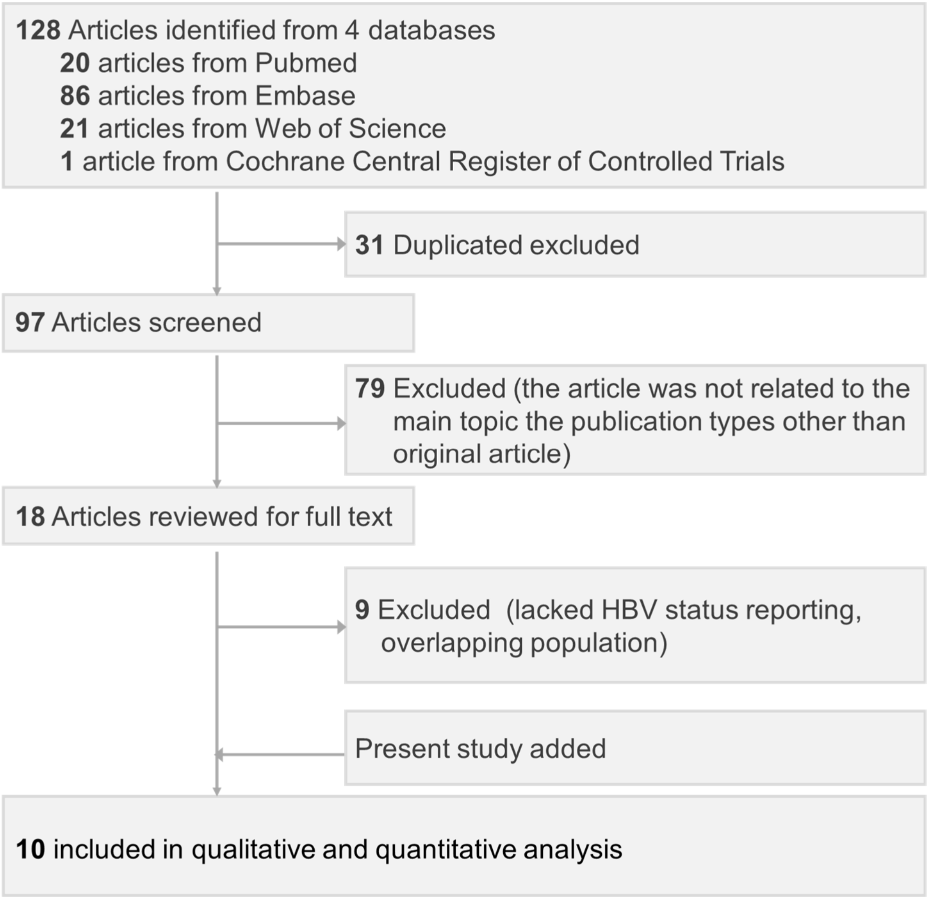

Figure S2. Publication bias analysis by using funnel plot for HBVr incidence rate of HBsAg-/HBcAb+ patients without prophylaxis.

(Egger's test: p=0.56)

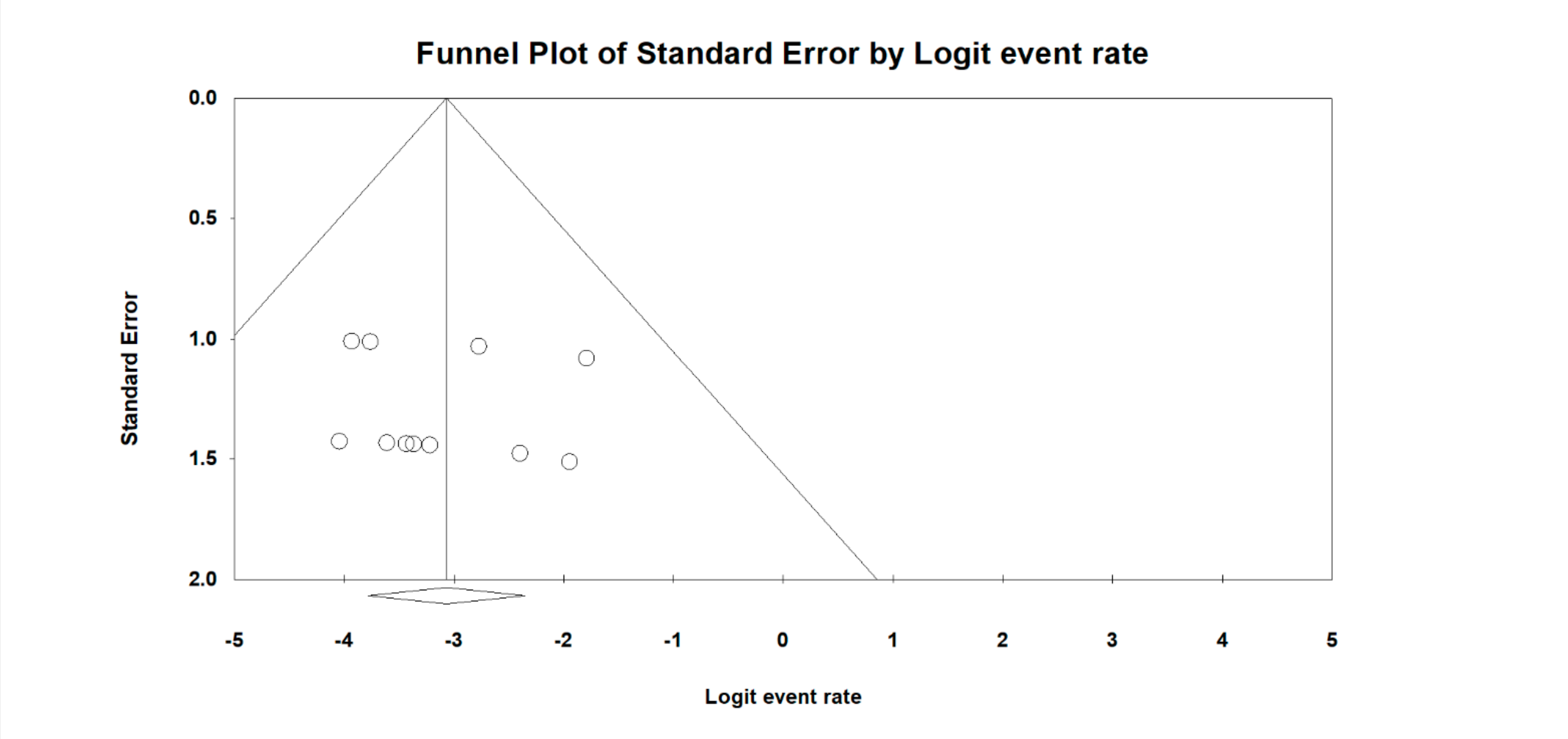

Figure S3. Sensitivity analysis

A. HBsAg + patients without prophylaxis

Study name	Point	Lower limit	Upper limit
Chiu 2013	0.180	0.064	0.414
Ting 2018	0.193	0.068	0.438
Qin 2022	0.217	0.092	0.430
Lu 2024	0.224	0.096	0.441
Present study	0.234	0.100	0.456
Pooled	0.212	0.094	0.410

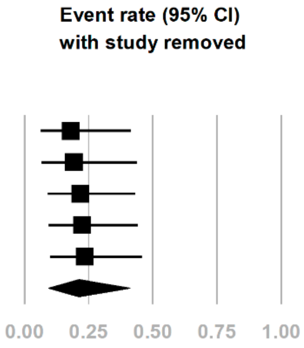

B. HBsAg−/HBcAb+ patients without prophylaxis

Study name	Point	Lower limit	Upper limit
Chiu 2013	0.042	0.020	0.083
Snast 2017	0.045	0.022	0.090
AlMutairi 2018	0.047	0.023	0.094
Ting 2018	0.049	0.023	0.100
Klujszo 2022	0.043	0.021	0.085
Megna 2022	0.043	0.020	0.087
Qin 2022	0.046	0.022	0.091
Gargiulo 2022	0.045	0.022	0.090
Lu 2024	0.046	0.023	0.092
Present study_a	0.038	0.018	0.078
Present study_b	0.050	0.024	0.102
Present study_c	0.042	0.020	0.083
Pooled	0.044	0.022	0.087

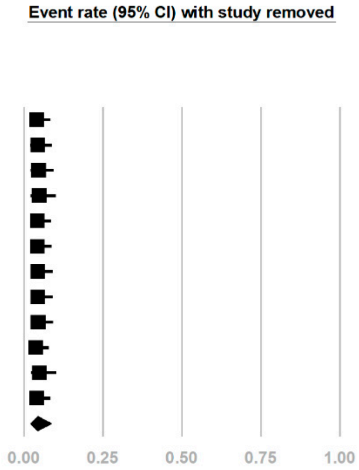

Supplement: Supplementary file 1 [file viruses-17-00042-s001.zip › viruses-3365714-supplementary.pdf]
